# Application of QuEChERS for Determining Xenobiotics in Foods of Animal Origin

**DOI:** 10.1155/2017/2603067

**Published:** 2017-12-24

**Authors:** Coralia V. Garcia, Ahmed Gotah

**Affiliations:** Department of Food Science and Technology, Keimyung University, Daegu 42601, Republic of Korea

## Abstract

The use of pesticides and veterinary drugs results in the appearance of residues of xenobiotics in foods. Thus, several methods have been developed for monitoring them; however, most are tedious and expensive. By contrast, the QuEChERS (Quick, Easy, Cheap, Effective, Rugged, and Safe) methodology involves a microextraction that yields small samples and has been applied for the analysis of various xenobiotics including pesticides, antibiotics, and mycotoxins. QuEChERS has shown advantages over other techniques including fast sample preparation, reduced needs for reagents and labware, and versatility. This approach allows the simultaneous determination of pesticides with various polarities and volatilities and can be easily modified for the analysis of a wide range of xenobiotics in various matrices including animal products rich in fat. Nevertheless, to attain high recoveries, the extraction, cleanup, and concentration steps have to be optimized according to the target compounds and matrix. Hence, QuEChERS is a promising and environmentally friendly methodology for the high-throughput routine analysis of xenobiotics in animal products. This review focuses on the application of QuEChERS to foods of animal origin and describes recent developments for the optimization of the analysis of veterinary drugs, pesticides, polycyclic aromatic hydrocarbons, and other compounds of concern.

## 1. Introduction

**Figure 1:**
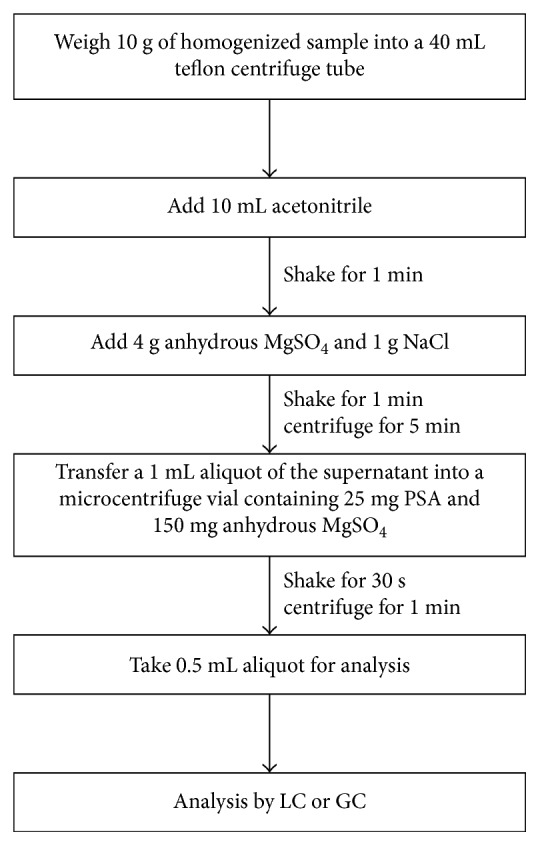
Flowchart of the QuEChERS method (based on [1] and [2]).

**Figure 2:**
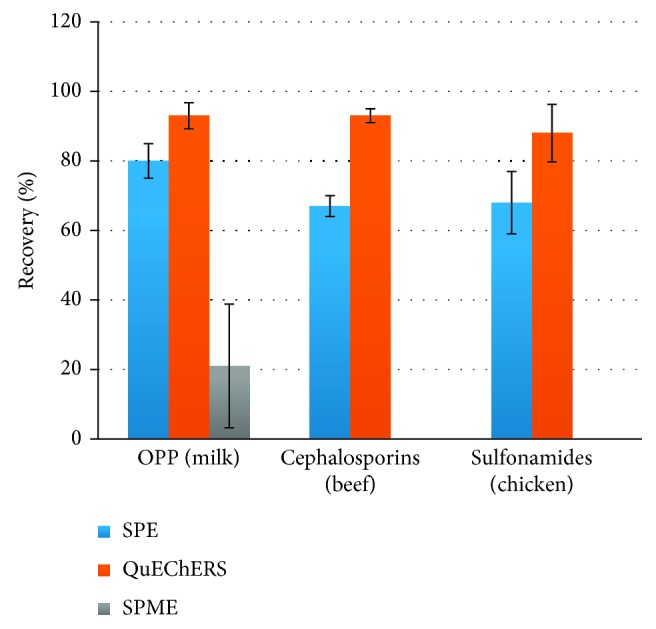
Comparison of the recoveries obtained using QuEChERS with those obtained with other extraction methods (OPP: organophosphate pesticides, SPE: solid-phase extraction, SPME: solid-phase microextraction) for selected classes of compounds according to published data [3–5].

The QuEChERS (Quick, Easy, Cheap, Effective, Rugged, and Safe) methodology was reported for the first time in 2003 by Anastassiades and coworkers as an alternative to traditional pesticide analysis [1]. QuEChERS involves a microscale extraction with acetonitrile combined with dispersive solid-phase extraction (d-SPE) using primary secondary amine (PSA) or other sorbents for purifying the extract (Figure 1). This methodology shows advantages over traditional pesticide analysis as it requires only a small amount of reagents, and sample isolation and cleanup are achieved in a single step instead of a series of time-consuming solvent extractions. Furthermore, acetonitrile is the preferred solvent for QuEChERS instead of toxic organochlorine solvents, making QuEChERS more environmentally friendly. In addition, the acetonitrile extracts produced are versatile and can be analyzed by liquid or gas chromatography, and recoveries are generally high (Figure 2). Thus, the methodology has been modified for analyzing various pesticides including pyrethroids, organochlorine, and phosphorous pesticides in various food matrices [2].

QuEChERS was originally developed as a multiclass residue method for determining pesticides in fruits and vegetables [1]. Because fruits and vegetables have high water content and are low in fat, application of QuEChERS for pesticide analysis in other food matrices requires modifications. QuEChERS was adapted for analyzing pesticides in cereals by adding water to the dry ground sample, producing slurry for extraction [6], whereas C_18_ was added for removing interfering lipids in oily samples like flaxseeds and peanuts [7].

Because pesticides are not the only xenobiotics in foodstuffs, analysis of veterinary drugs, mycotoxins, and other contaminants cannot be neglected. So far, QuEChERS has been predominantly applied for the analysis of contaminants in foods of plant origin. In addition to pesticides in various fruits and vegetables [2], acrylamide in potato chips, peanut butter, and chocolate [8] and aflatoxins in noodles [9] have been determined using QuEChERS.

QuEChERS has been demonstrated to be an effective and versatile technique and has also been recently applied for the analysis of various xenobiotics in animal products. The complexity of animal tissues and milk as well as the presence of fat are challenges to overcome for the successful analysis of xenobiotics in such matrices. Although several matrix-specific methods are reported here, it is noteworthy that a simple, modular QuEChERS method was developed for analyzing pesticides in animal products with various contents of fat, attaining adequate recoveries that could make it a good option for routine sample screening; this method involved extraction of the sample in acetonitrile, followed by hexane extraction in the presence of a QuEChERS salt and cleanup by SPE (C_18_) [10].

Therefore, this review presents a compilation of examples of the application of QuEChERS to the analysis of xenobiotics including pesticides, mycotoxins, and veterinary drugs in animal products such as meat, milk, eggs, and honey (Table 1). Because new studies on this topic are increasingly appearing in the literature, the list is not exhaustive but focuses on major products and xenobiotics of concern as well as reports from the previous 5 years. It is expected that this review will provide information about the use of QuEChERS as an alternative technique for the analysis of various contaminants in food products of animal origin. In addition, considerations about solvent and sorbent selection are presented.

## 2. Application of QuEChERS to Foods of Animal Origin

### 2.1. Meat

Veterinary drug residues are xenobiotics of concern in meat products. These drugs include antibiotics, antiparasitic agents, compounds that stimulate muscle production, growth hormones, and anti-inflammatories. Residues of pesticides and tranquilizers can also be found in meat, and dangerous compounds like nitrosamines can be present in processed meat products. Therefore, monitoring of these substances in meat is crucial.

The first report of QuEChERS applied to animal tissues described the analysis of β-lactam antibiotics in bovine kidneys [49]. β-Lactams include penicillins and cephalosporins and are used to treat bacterial infections as well as to promote weight gain in farm animals. The presence of antibiotic residues in meat is also of concern as it may trigger adverse reactions in hypersensitive individuals [50]. Thus, effective methods are required to monitor these compounds and ensure that they are not present in foods at levels above those considered safe. In the study by Fagerquist et al. [49], QuEChERS extraction was achieved using water and acetonitrile, and C_18_ was added to remove interfering fats; subsequently, the extract was analyzed by LC-MS/MS. LC-MS/MS is the technique of choice for detecting drug residues because it can determine compounds with high molecular mass as well as polar, nonvolatile, and heat-sensitive compounds [11]. Another report described the analysis of cephalosporins in beef muscle by LC-MS/MS after QuEChERS extraction using C_18_, PSA, and MgSO_4_ as sorbents for d-SPE [3]; the proposed QuEChERS method was comparable to SPE and sensitive enough to comply with European Union regulations.

Antiparasitic drugs are administered to farm animals to control roundworm infections. Some common anthelmintic drugs include benzimidazoles, macrocyclic lactones, and amino-acetonitrile derivatives. As with antibiotics, residues of these compounds can show up in meat and milk and should thus be monitored. Kinsella et al. [11] reported a modified QuEChERS method for detecting the anthelmintic monepantel and its sulfone metabolite in both sheep meat and goat's milk. Their method added a concentration step to increase sensitivity, and samples were analyzed by UHPLC-MS/MS. The method showed fast separation (13 min) and good recoveries (108%-109%) for both monepantel and its metabolite.

QuEChERS can also be used to detect the presence of residues of banned feed additives that could appear in animal products. Ractopamine is a β-agonist used as a feed additive for finishing pigs as it promotes the production of muscle instead of fat. Nevertheless, β-agonists may produce toxic effects on the cardiovascular system [51], and ractopamine has been banned in Europe and parts of Asia. A Brazilian team quantified ractopamine in pork by QuEChERS-LC-MS/MS [16] in only 4.5 min, which represents substantial time savings compared to conventional ractopamine analysis, which is performed by liquid-liquid extraction followed by derivatization and GC-MS analysis [52] or liquid-liquid extraction followed by HPLC or LC-MS [53, 54]. In addition to being fast, the QuEChERS method of Valese et al. [16] achieved adequate recoveries (70%–110%) by using acidified (0.1% acetic acid) acetonitrile for the extraction and a low LOD (1.5 µg/kg). Ten β-agonists including ractopamine were simultaneously determined in beef and goat meat by a similar QuEChERS-LC-MS/MS method, which used a DVB-NVP-SO_3_Na sorbent for d-SPE [55]; in this study, a preconcentration step was performed to enhance sensitivity, achieving an even lower LOD (0.3 µg/kg) than that of Valese et al. [16].

As previously mentioned, the QuEChERS methodology was originally developed for the analysis of pesticide residues in vegetables and fruits and has been successfully applied for determining pesticides in various foodstuffs. Examples of QuEChERS application for determining pesticide residues in meat include the analysis of organochlorine pesticides and pyrethroids in sheep meat [15] and multiclass pesticide residues in beef and bovine fat [12]. Samples in these studies were analyzed by GC coupled to MS or ECD, and fat samples required partitioning in hexane prior to analysis. Recoveries were in the range 70%–129% with RSD < 20%.

In addition to organochlorine pesticides, polychlorinated biphenyls (PCBs) are other type of persistent organic pollutants that may contaminate meat. Even though PCBs were banned decades ago, their residues still appear in food products because of their stability and lipid-soluble properties; these compounds have harmful effects on human health including carcinogenicity, neurotoxicity, and developmental disorders in children. A QuEChERS-GC-MS method for determining PCBs in meat was optimized and successfully applied for analyzing processed meat products [14]; the method used ultrasonication to improve extraction efficiency and achieved LOQs below 1.3 ng/g (the MRL established by the European Community is 40 ng/g) and high recoveries, representing a cheaper and environmentally friendly alternative for the analysis of PCBs.

Some harmful compounds are formed during meat processing or cooking. This is the case of nitrosamines, carcinogens produced when cooking cured meats. The US Department of Agriculture Food Service and Inspection Service (USDA-FSIS) has established a method for determining nitrosamines using supercritical fluid extraction followed by GC-thermal energy analysis (GC-TEA) [56]; however, this is a costly and tedious methodology, and better alternatives are needed. A new QuEChERS-GC-MS/MS methodology was developed for determining nitrosamines in cooked bacon, achieving good recoveries (70%–120%) with low RSD (<20%) [13], and was thus proposed to the USDA-FSIS as an efficient alternative for the monitoring of these compounds in bacon, an important source of exposure to nitrosamines in the American population.

### 2.2. Poultry

As with cattle, veterinary drugs are administered to chicken and other birds raised for food to fight infections and promote growth. Thus, residues of these substances must be monitored to ensure the safety of the meat and avoid potential harmful effects among consumers.

**Figure 3:**
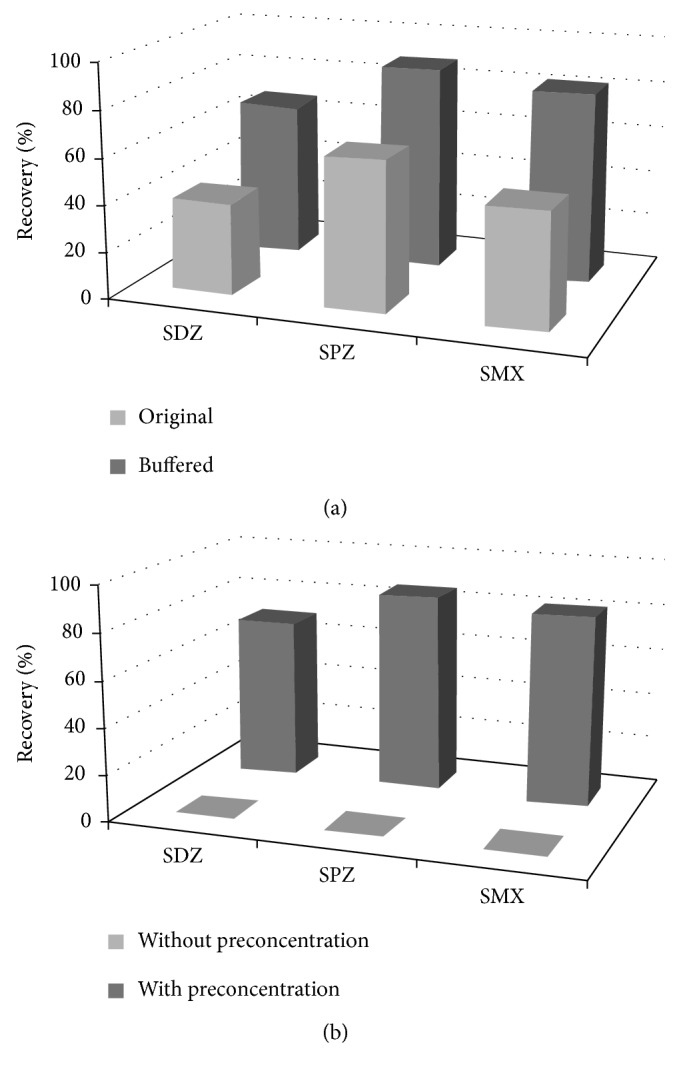
Effects of (a) using the original or buffered QuEChERS and (b) concentrating the extract on the recovery of sulfonamides (sulfadiazine (SDZ), sulfamethoxypyridazine (SPZ), and sulfamethoxazole (SMX)) from chicken. Taken from [4].

Sulfonamides, also known as sulfa drugs, are synthetic antibiotics used to combat bacterial infections. Nevertheless, allergic reactions to these drugs are common, and residues should be monitored to ensure they are not present at levels above those considered safe. A QuEChERS-HPLC-DAD method for detecting sulfonamides in chicken breast was reported in 2013; the method attained LOQs below the MRL established by the European Community (100 µg/kg) and recoveries above 70% [4] by using buffering salts and concentrating the extract before analysis (Figure 3). A similar study used HPLC/FLD and Z-Sep^+^ as a sorbent instead [57], also achieving LOQs below the established MRL, but the recoveries for some sulfonamides were slightly lower than those of Machado et al. [4]. A study using QuEChERS-LC-MS/MS described the simultaneous determination of 20 veterinary drug residues including several antibiotics and anthelmintics in chicken breast in less than 8.5 min [20].

Bird influenza is a serious problem that not only causes economic losses but also puts human health at risk. In the past, antiviral drugs were administered to chickens to protect them against influenza; however, the risk of resistance development by the influenza viruses, which would render drugs like Tamiflu ineffective, motivated several countries including the US and China to ban the use of antiviral drugs in poultry. Nevertheless, illegal use of antivirals in chicken still occurs, making it necessary to develop a method to detect residues of these drugs in chicken. Even though several methods for this purpose already exist, they generally require derivatization and tedious extraction procedures. A simultaneous determination of 14 antiviral drugs in chicken meat using QuEChERS-UPLC-MS/MS was reported [19]; the drugs monitored included antiviral drugs, antiherpes drugs, and an immunomodulator, and separation was achieved in less than 11 min. Another report [18] described the optimization of a QuEChERS-UHPLC-LTQ Orbitrap MS method for detecting the antivirals amantadine and rimantadine in chicken breast.

### 2.3. Milk

Milk can be contaminated with pesticides when cows consume contaminated feed or water, and administration of veterinary drugs to the animals can result in the appearance of residues of antibiotics, hormones, anthelmintics, and other drugs in both milk and meat. Milk is an important component in the diet of children; hence, contamination with xenobiotics is of great concern. Milk is an emulsified fatty product; thus, both hydrophilic and hydrophobic xenobiotics can be present in it, making multiresidue analysis challenging. Therefore, an optimized QuEChERS methodology would save on cost and time when analyzing xenobiotics in milk. Response surface methodology was used to optimize the amount of sorbents (NaOAc, PSA, and C_18_) used in a QuEChERS-GC-ECD method for the simultaneous determination of 14 pesticides in milk, achieving recoveries >82% for hydrophilic pesticides, although recoveries of four lipophilic pesticides were <80% [24]. In another study [25], PSA and ZrO_2_-based sorbents were used to purify the QuEChERS milk extracts before HPLC-DAD analysis for quantification of 30 pesticides, achieving a negligible matrix effect, adequate recoveries (70%–100%), and LOQs below the MRLs set by European regulations.

In addition, some QuEChERS-based methods for the analysis of veterinary drugs in milk have focused on particular compounds. Determination of the anthelmintic monepantel in milk using QuEChERS was reported by Kinsella et al. [11]. Another report focused on the determination of sulfonamides in milk [27], achieving a combined LOQ below the MRL set by the European Union (100 µg/kg).

The presence of hormone residues in milk is of concern because they have been associated with precocious puberty, breast cancer, and prostate cancer. Such residues occur by the, sometimes illegal, administration of hormones to cows to stimulate milk production. Hormone residues can be detected by ELISA; however, false positives are an issue. The low concentrations at which they occur and binding to proteins also hinder their detection by analytical equipment. A report described using PSA and ZnO nanoparticles as QuEChERS sorbents for the analysis of anabolic steroid and β-agonist residues in milk, using UHPLC-MS/MS [22]; application of the method to samples from Chinese markets revealed the presence of methyltestosterone, a banned substance, and other steroids. Another group of researchers used a combination of salts (anhydrous MgSO_4_, NaCl, sodium citrate, and sodium citrate dibasic sesquihydrate) to cleanup cow, goat, and buffalo milk samples for the analysis of steroid hormones, using UPLC-TOF-MS for their determination [26]; application of this method to several Chinese milk samples revealed the presence of high levels of progesterone.

Aflatoxins are dangerous carcinogenic mycotoxins that can contaminate grains, oilseeds, and spices and can also appear in milk if the cow had consumed contaminated feed. QuEChERS proved to be useful for the analysis of aflatoxins in milk, using UPLC-MS/MS for their detection [58]; application of the optimized method revealed the presence of aflatoxins M1 and M2 in several powdered milk samples from Brazil, although at concentrations below the established maximum tolerance level (MTL).

**Figure 4:**
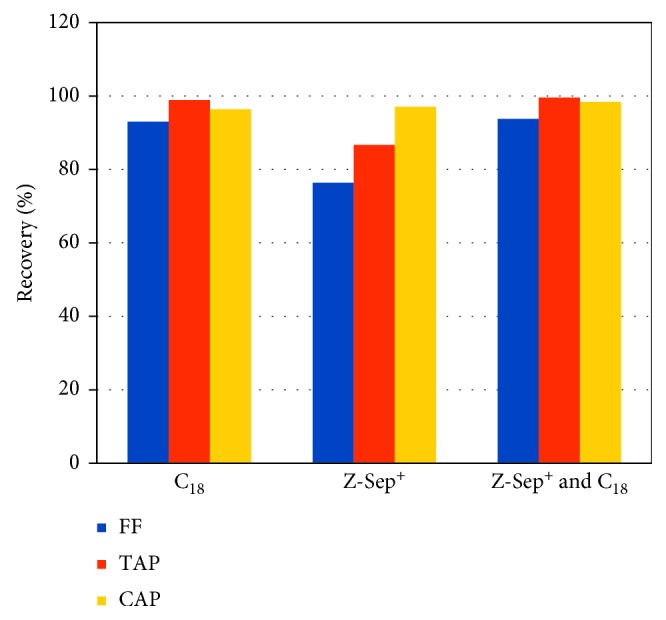
Effect of sorbent on the recoveries of florfenicol (FF), thiamphenicol (TAP), and chloramphenicol (CAP) from milk samples extracted by QuEChERS, according to data in [21]. Sorbents were C_18_ (350 mg), Z-Sep^+^ (500 mg), and Z-Sep^+^ (500 mg) combined with C_18_ (350 mg).

QuEChERS has also been used in combination with other techniques to achieve purer extracts, facilitating the analysis of trace compounds. QuEChERS was used as a pretreatment step prior to capillary LC-MS/MS for determining chloramphenicol, thiamphenicol, and florfenicol in milk [21]. Chloramphenicol is a banned antibiotic in cattle in the European Union, which has set a minimum required performance limit (MRPL) of 0.3 ng/g. To develop their modified QuEChERS method, Liu et al. [21] tested various sorbents, among which a combination of Z-Sep^+^ and C_18_ yielded excellent recoveries (96%–100%) (Figure 4), minor matrix effects, and low LODs (≤0.045 ng/g) representing a promising technique for the analysis of amphenicol traces in milk and similar products. In another study, QuEChERS was performed prior to dispersive liquid-liquid microextraction based on the solidification of the floating organic droplet (DLLME-SFO) method for determining organophosphorous pesticides in milk [5]; the method used 1-dodecanol for the extraction instead of the organochlorine solvents generally used for DLLME, being more environmentally friendly and also allowing for easy and rapid analysis by GC.

### 2.4. Eggs

Eggs may contain many of the same xenobiotics found in chicken including antibiotics, hormones, and pesticides; nevertheless, the MRLs may differ as some products that are allowed in birds raised for meat are banned in egg-laying hens. Moreover, eggs are a complex matrix rich in protein and lipids; their lipoproteins may bind to analytes, and foam may be produced during extraction, making sample cleanup and analysis challenging.

Huertas-Perez et al. [57] used acidified acetonitrile and PSA as a sorbent for the analysis of sulfonamides in eggs by QuEChERS-HPLC-FLD. In contrast with chickens raised for meat, these antibiotics are banned in egg-laying hens. Postcolumn photochemical derivatization was performed to increase the sensitivity of the method, achieving LODs < 9 µg/kg, except in the case of sulfachloropyridazine. Acidified acetonitrile was also used to improve the extraction of polar pesticides in eggs, and clean samples were attained using a commercial sorbent (Bond Elut d-SPE universal kit) [32]; the method yielded good recoveries (70%–108%) and low LOQs of 0.3–6 ng/g, which are well below the MRLs (≥50 ng/g) of the pesticides analyzed.

**Figure 5:**
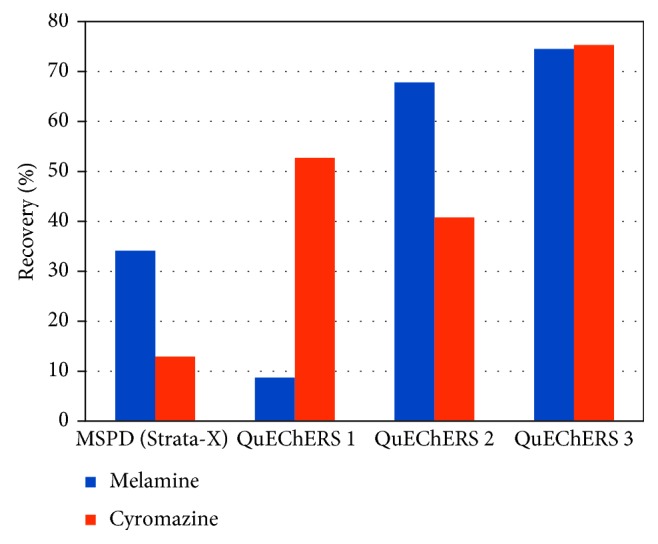
Effects of solvent on the recoveries of melamine and cyromazine from eggs, using a commercial QuEChERS sorbent for fatty samples (QuEChERS 1 = 2 mL MeCN, QuEChERS 2 = 2 mL MeOH, and QuEChERS 3 = 1 mL MeOH + 1 mL MeCN) and comparison with matrix solid-phase dispersion (MSPD), according to data in [29].

Melamine is a nitrogenated compound used in the manufacture of plastics and has been illegally added to milk to increase its protein content, with nefarious effects on human health as melamine exhibits renal toxicity. Melamine can also be generated as a by-product of the metabolism of cyromazine in hens. Cyromazine is an insecticide added to the feed of egg-laying hens to prevent maggot infestations in their manure; however, residues of both the insecticide and its melamine metabolite require careful monitoring because of their toxicity. Melamine and cyromazine have been extracted using QuEChERS with acidified acetonitrile or a methanol-acetonitrile mixture and determined by HPLC-DAD [29] or LC-MS/MS [30]. Between these two methods, the recoveries (83%–105%) obtained by the method of Wang et al. [30] were slightly higher, but the LOQ obtained by Tsartsali and Samanidou [29] was substantially lower (2.5 versus 8 µg/kg); thus, considering the performance of the methods and cost of the equipment, QuEChERS-HPLC-DAD is a valid option for determining melamine in eggs. In this report [29], using a combination of methanol and acetonitrile yielded substantially higher recoveries than those obtained with other solvents (Figure 5), demonstrating that solvent selection is crucial for a successful QuEChERS extraction.

Mycotoxins can contaminate eggs when hens ingest contaminated feed. Because mycotoxins are powerful carcinogens, their presence in food products should be carefully monitored. Mycotoxins have been determined in eggs using QuEChERS-UPLC-MS/MS either with a cleanup step [28] or without it [31]. The method of Li et al. [28] achieved slightly higher recoveries (85%–115%), but that of Frenich et al. [31] achieved lower LOQs (1-2 µg/kg versus 3–15 µg/kg) for aflatoxins and faster separation. Nevertheless, both proved to be applicable to real samples, detecting residues of aflatoxins in at least one egg sample.

### 2.5. Fish

The contamination of aqueous ecosystems with pesticides, pharmaceutical waste, heavy metals, and other substances has resulted in the accumulation of xenobiotics in fish, representing a risk to human health. Therefore, effective multiresidue analysis methods are necessary to monitor the presence of these contaminants in fish. Because of their speed, robustness, and environmental friendliness, QuEChERS-based methods have been used to analyze various contaminants in fish, with excellent results.

The most important contaminants in fish are persistent organic pollutants such as organochlorine pesticides and polychlorinated biphenyls, which accumulate in the adipose tissue and have negative effects on human health. A modified QuEChERS method using CaCl_2_ and PSA and a freezing-out step for removing fats from fish tissue attained good GC-MS recoveries (70%–115%) for both organochlorine pesticides and polychlorinated biphenyls in tilapia, although recoveries in salmon were lower likely because of the high fat content of this fish [34]. Nevertheless, using a mixture of acetonitrile/tetrahydrofurane in this method instead of acetonitrile alone enhanced recoveries of most compounds from salmon. Another group of researchers opted for QuEChERS followed by the DLLME-SFO technique for cleanup of tilapia samples for determining organochlorine pesticides by GC-ECD, achieving recoveries > 88% [41].

**Figure 6:**
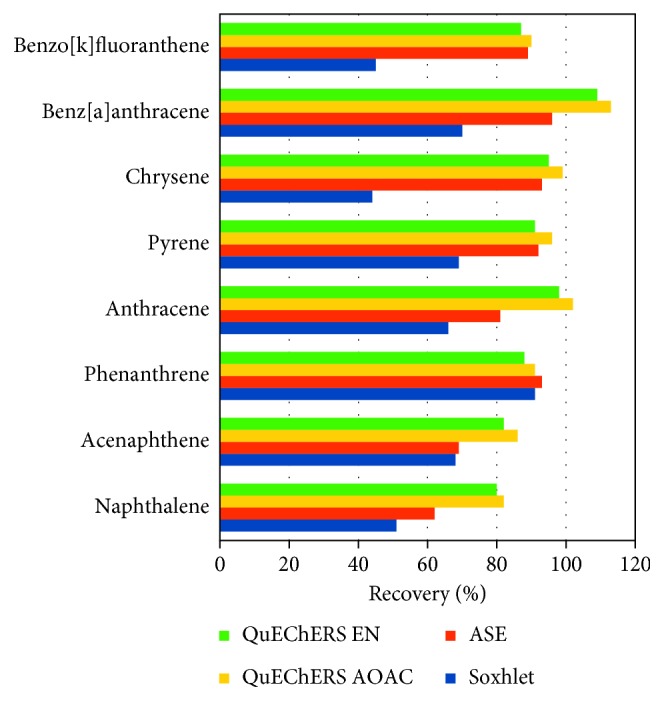
Recoveries of selected polycyclic aromatic hydrocarbons (PAHs) from fish, attained using QuEChERS with AOAC extraction salt (QuEChERS AOAC), QuEChERS with EN extraction salt (QuEChERS EN), Soxhlet with hexane, and accelerated solvent extraction (ASE) with CH_2_Cl_2_ : MeCN (9 : 1), according to data in [36].

Polycyclic aromatic hydrocarbons (PAHs) are another group of important contaminants in fish. PAHs are combustion by-products that can accumulate in the adipose tissue and exhibit mutagenic and carcinogenic effects. QuEChERS-GC/MS has been successfully used to determine PAHs in fatty fish including herring and salmon [35, 36]. Forsberg et al. [36] used ethyl acetate, acetone, and isooctane instead of the more common acetonitrile for the QuEChERS extraction and attained overall recoveries almost 40% higher than those achieved by the traditional Soxhlet method. A comparison of the recoveries of selected PAHs obtained with QuEChERS and other methods as reported by [36] is shown in Figure 6. Moreover, like in meat, QuEChERS proved useful for determining PCBs in fish, also attaining low LOQs (<1 ng/g) [39].

Veterinary drugs are used in fish farming because of the intensity of such operations, in which large numbers of fish are raised in a small space, becoming prone to infection by microorganisms and parasites. QuEChERS has been applied for the determination of malachite green [59] and quinolones [40] in fish, using LC-MS/MS and UPLC-FLD, respectively.

For multiclass pesticide analysis in fish muscle, a novel dual d-SPE cleanup step was introduced [38]. Briefly, after partition of the extract with salts, a first cleanup was performed using PSA, SAX, NH_2_, and MgSO_4_, and the obtained supernatant was subjected to a second cleanup with C_18_ followed by freezing-out. As a result, cleaner samples and increased sensitivity was attained, with GC-MS recoveries of 70%–120% and LOQs in the range 0.004–0.009 mg/kg. In addition, multiclass analysis of pharmaceuticals in bivalves attained LOQs < 100 ng/g and revealed the presence of salicylic acid, demonstrating that metabolites of pharmaceuticals can contaminate aquatic species [42].

In addition to contaminants derived from human activities, paralytic shellfish poisoning toxins, which are produced by algae and cause severe food poisoning, have also been determined using QuEChERS [43].

### 2.6. Honey

Honey is a complex mixture of sugars and waxes that vary according to the nectar used by the bees to create it, making its analysis challenging. Pesticide contamination of nectar and honey is not only of concern to human health but also may be associated with the phenomenon of disappearance of hives. Therefore, monitoring of pesticide residues in honey is of interest to ensure its safety to consumers and to determine if pesticides are being accumulated and affect beehives. A multiresidue QuEChERS-based method for analyzing pesticides in honey was proposed [45]; the honey was heated and dissolved in Na_2_EDTA prior to extraction with acetonitrile and d-SPE, and the resulting extract was analyzed by GC-ECD. Another method used UPLC-MS/MS for determining pesticides after QuEChERS extraction, which involved dissolving the honey in water and using a mixture of acidified acetonitrile/ethyl acetate [46]. The method of Tette et al. [46] did not require heating the honey samples and achieved a faster separation than that of Orso et al. [45], although the LODs were comparable.

Among pesticides, neonicotinoids are of particular concern because of their harmful effects on bees and their possible role in bee colony collapse. QuEChERS extraction of neonicotinoids from honey was achieved by dissolving honey samples in water and extracting them with acetonitrile, using SPE citrate sorbent for d-SPE; the clean extracts were then analyzed by UPLC-MS/MS, achieving LODs < 2.5 µg/kg [60].

The presence of antibiotic residues in honey is also of concern. For analyzing nitrofurans and nitroimidazoles in honey, samples were derivatized with acid, QuEChERS extraction was performed without sorbent addition, and the extract was evaporated and reconstituted prior to LC-MS/MS analysis; the validated method complied with European guidelines [44].

Because honey can also be simultaneously contaminated with various xenobiotics, a multiresidue method able to determine various compound classes would be useful; such a method was recently reported, using a combination of conventional QuEChERS and SPME with both LC-MS/MS and GC-MS/MS for determining more than 100 compounds between pesticides, PAHs, and PCBs [47].

In addition to contaminants derived from human activities, it is known that honey may become contaminated with natural toxins like alkaloids from various plant species, which is of concern particularly when children consume it; thus, QuEChERS was recently applied to determine both pyrrolizidine and tropane alkaloids in honey [48].

## 3. Final Considerations

The QuEChERS methodology has been successfully applied for the analysis of various contaminants in food products of animal origin, yielding high recoveries that are comparably higher than those obtained with other techniques, while decreasing the needs for time and reagents. Nevertheless, to ensure the success of this technique, solvent and sorbent selection is of critical importance. As shown in Table 1, acetonitrile has been the solvent of choice for QuEChERS extractions because it can be easily separated from water by salt partitioning, tends to extract less interfering substances compared with other solvents, and the produced extract can be analyzed by gas or liquid chromatography [2]. Acetonitrile has been used alone, mixed with water, or modified by adding acids. Although sample : solvent ratios vary, ratios of 1 : 2 are common. Greater solvent volumes tend to yield higher recoveries, up to a certain point [23, 55]; thus, the volume used also has to be optimized. Acetonitrile added with acetic acid was found to improve the extraction of ractopamine in pork [16] and antibiotics and antiviral compounds in chicken [4, 19], whereas acetonitrile acidified with formic acid was used for extracting quinolones from fish [40]. High-fat samples have been extracted with other solvents such as hexane and acetone followed by acetonitrile extraction, or a combination of solvents capable of dissolving fat like the acetone/ethyl acetate/isooctane mixture used to extract PAHs from salmon [36]. Partitioning is achieved using NaCl and MgSO_4_ in most cases, although buffered salts have been used for extracting contaminants such as nitrites from bacon [13], veterinary drugs from chicken [20], steroids from milk [26], and PAHs from salmon [36]. Subsequently, the extracts are cleaned up by d-SPE, for which the sorbents must be chosen depending on the characteristics of the matrix and compounds of interest. MgSO_4_ and PSA are common sorbents used for various contaminants and matrices, whereas C_18_ is added for the cleanup of high-fat samples including milk, bacon, red meats, eggs, and fish. Novel sorbents such as Z-Sep and Z-Sep^+^, which are a combination of C_18_ and ZrO_2_, have been used in combination with C_18_ and PSA to improve the cleanup of high-fat samples such as bacon and milk [13, 21, 25], and they have been promoted as a convenient replacement for the traditional PSA + C_18_ combination used for such samples. Fat removal can also be achieved by freezing-out of samples, although to obtain cleaner extracts, this step is preceded by d-SPE as described above. To overcome the disadvantage of producing extracts with low concentrations of compounds, evaporating and redissolving the sample in a small amount of solvents is commonly done. One of the problems reported for QuEChERS extracts of lipophilic compounds such as organochlorine pesticides is their low enrichment factor; thus, QuEChERS has been combined with the DLLME-SFO technique to produce cleaner and more concentrated extracts yielding higher recoveries, an example being a report on the analysis of organochlorine pesticides in fish samples [41]. Nanotechnology has also appeared in the development of QuEChERS methods, with a report describing the use of ZnO nanoparticles and PSA for purifying milk and honey extracts containing steroids [22]. However, this d-SPE step has been omitted in some reports without negative effects, including the analysis of mycotoxins in eggs [31] and nitrofuran metabolites in honey [44], which may be due to the limited number of target compounds in these cases.

## 4. Conclusion

The QuEChERS methodology has demonstrated to be applicable to the analysis of various xenobiotics including pesticides, hormones, toxins, and antibiotics in animal matrices such as meat, milk, eggs, and honey. QuEChERS exhibits advantages over traditional methods because of its speed, efficiency, and environmental friendliness. Recoveries obtained using QuEChERS tend to be comparable or even higher than those obtained by solvent extraction or SPE, while reducing the need for solvent as well as the analysis time. QuEChERS has also proven to be applicable to the analysis of compounds that are of particular concern in recent times, such as melamine and ractopamine. Therefore, it is expected that new modifications to the QuEChERS methodology will continue to be developed to facilitate the analysis of more veterinary drugs and other xenobiotics in animal products, and the methodology may become a favored choice for the monitoring of these substances in the future.

## Figures and Tables

**Table 1:** Summary of applications of QuEChERS for the analysis of xenobiotics in animal products.

Matrix	Analytes	QuEChERS method	Analytical method	Ref.
Goat's milk and sheep muscle	Monepantel and its sulfone metabolite	Milk (10 g) was extracted with MeCN (12 mL) in the presence of MgSO_4_ and NaCl, followed by d-SPE using MgSO_4_ and C_18_. Homogenized meat (10 g) was extracted with MeCN (10 mL); MgSO_4_ and NaCl were added, and the supernatant was cleaned up with MgSO_4_ and C_18_. Extracts were concentrated before analysis.	UPLC-MS/MS	[11]

Beef muscle	Cephalosporins	Homogenized beef (4 g) was extracted with 15 mL of MeCN/water (80 : 20); d-SPE was performed using C_18_, PSA and MgSO_4_. The sample was dried and reconstituted before analysis.	LC-MS/MS	[3]

Meat and bovine fat	Chlorfenvinphos, fipronil, and cypermethrin	For the meat, 2 g of homogenized sample was extracted in 4 mL of MeCN in the presence of MgSO_4_ and NaCl, followed by d-SPE with C_18_, PSA, and MgSO_4_. For the fat, 1 g of homogenized sample was extracted in 5 mL of hexane + 10 mL of water + 10 mL of MeCN in the presence of MgSO_4_ and NaCl, followed by d-SPE with PSA and MgSO_4_.	GC-MS	[12]

Bacon	Nitrites	Comminuted cooked bacon (5 g) was extracted with 10 mL of MeCN/water (1 : 1) in the presence of HCO_2_NH_4_, followed by d-SPE using MgSO_4_, PSA, C_18_, and Z-Sep sorbents. Coextracted lipids were removed by hexane partitioning.	GC-MS/MS	[13]

Meat products	Polychlorinated biphenyls (PCBs)	Melted fat sample (1 g) was extracted with 7 mL of acetone. The supernatant was dried and collected using 2 mL of acetonitrile in the presence of PSA and MgSO_4_. The sample was dried and collected using isooctane before injection.	GC-MS	[14]

Sheep meat	Chlorinated hydrocarbon and synthetic pyrethroids	Homogenized meat (10 g) was extracted with 20 mL of MeCN in the presence of MgSO_4_ and NaCl, followed by d-SPE using PSA and MgSO_4_. Samples were dried and redissolved before analysis.	GC-ECD	[15]

Pork	Ractopamine	Ground pork sample (5 g) was extracted with 10 mL of 0.1% AcOH in MeCN, followed by cleanup using MgSO_4_, NaCl, C_18_, and PSA. Samples were dried and redissolved before analysis.	LC-MS	[16]

Pork	Methenamine	Homogenized sample (2 g) was extracted with 10 mL of MeCN in the presence of Na_2_SO_4_, followed by extraction with MeCN-saturated hexane. The MeCN was cleaned up with PSA.	LC-MS/MS	[17]

Chicken	Amantadine and rimantadine	Homogenized chicken meat (3 g) was extracted with 10 mL of 1% AcOH in MeCN in the presence of NaCl and MgSO_4_, followed by d-SPE using C_18_. The sample was dried and redissolved before analysis.	UHPLC-LTQ Orbitrap MS	[18]

Chicken	Antiviral drugs and relevant metabolites	Homogenized chicken meat (2 g) was extracted with 9 mL of 1% AcOH in MeCN + 1 mL of water. The supernatant was split into two portions for the analysis of different compounds and dried. One portion was redissolved in MeOH/water and the other in water. The extracts were cleaned up using PSA.	UPLC-MS/MS	[19]

Chicken	Veterinary drugs	Homogenized chicken meat (5 g) was extracted with 10 mL of 1% AcOH in MeCN: water (80 : 20) in the presence of Na_2_HCit·1.5H_2_O, Na_3_Cit·2H_2_O, and MgSO_4_, followed by d-SPE using PSA.	UHPLC-MS/MS	[20]

Chicken	Sulfonamides	Homogenized chicken meat (10 g) was extracted with 15 mL of 1% AcOH in MeCN in the presence of NaOAc and MgSO_4_, followed by d-SPE using PSA and MgSO_4_. The sample was concentrated before analysis	HPLC-DAD	[4]

Milk and honey	Chloramphenicol, thiamphenicol, and florfenicol	Sample (2 g) was extracted with 15 mL of 0.1% AcOH in MeCN in the presence of Na_2_SO_4_ and NaCl, followed by d-SPE using Na_2_SO_4_, C_18_, and QuE Z-Sep^+^. Samples were dried and reconstituted before analysis.	LC-MS/MS	[21]

Milk	Anabolic steroids and agonists	Milk (5 g) was extracted with 10 mL of 5% AcOH in MeCN in the presence of ZnO nanoparticles, followed by d-SPE using PSA. Samples were dried and redissolved before analysis.	UHPLC-MS/MS	[22]

Yogurt and milk	Herbicides (triazines and phenylureas)	Sample (5 mL) was adjusted to pH 7 and extracted with 8 mL EtOAc/hexane (1 : 1) in the presence of NaCl, followed by d-SPE using PSA. The extract was dried and redissolved before analysis.	HPLC-DAD	[23]

Milk	Pesticides	Milk (15 g) was extracted with 15 mL of 0.1% AcOH in MeCN in the presence of NaOAc and MgSO_4_, followed by d-SPE using MgSO_4_, PSA and C_18_.	GC-ECD and GC-MS	[24]

Milk	Pesticides	Milk (20 mL) was extracted with 16 mL MeCN in the presence of NaCl and MgSO_4_. The extract was dried and reconstituted, followed by d-SPE using PSA, Z-Sep, and Z-Sep Plus. The supernatant was dried and redissolved before analysis.	HPLC-DAD	[25]

Raw milk (cow, goat, and buffalo milk)	Steroid hormones	Milk (10 g) was extracted with 10 mL MeCN in the presence of NaCl, Na_2_HCit, Na_2_HCit·1.5H_2_O, and MgSO_4_, followed by d-SPE using MgSO_4_, PSA, and acidic alumina.	UPLC-QTOF-MS	[26]

Milk	Sulfonamides	Milk (10 g) was extracted with 10 mL of 1% AcOH in MeCN + 10 mL 0.1 M EDTA in the presence of NaOAc and MgSO_4_.	LC/MS/MS	[27]

Eggs	Aflatoxins B1, G1, B2, G2, zearalenone, and its metabolites	Homogenized eggs (2 g) + 2 mL water was extracted with 10 mL of 1% AcOH in MeCN in the presence of MgSO_4_ and NaCl, followed by d-SPE using MgSO_4_ and C_18_. The extract was dried and redissolved before analysis.	UPLC-MS/MS	[28]

Eggs	Melamine and cyromazine	Homogenized egg yolk (0.5 g) was extracted with 1 mL MeCN + 1 mL MeOH in the presence of QuEChERS sorbent for fatty samples. The extract was dried and redissolved before analysis.	HPLC-DAD	[29]

Eggs	Melamine and cyromazine	Homogenized egg sample (1 g) was extracted with 5 mL MeCN + 0.1 M HCl (99.5 : 0.5), followed by d-SPE using MgSO_4_ and GCB. The extract was dried and redissolved before analysis.	LC-MS/MS	[30]

Eggs	Mycotoxins	Homogenized egg (2 g) was extracted with 10 mL of MeOH/water (80 : 20) with 1% AcOH, Na_2_SO_4_, and NaOAc, without d-SPE.	UHPLC-MS/MS	[31]

Eggs and egg products	Pesticides	Homogenized egg (5 g) was extracted with 1% AcOH in MeCN (15 mL) in the presence of MgSO_4_ and NaOAc, followed by d-SPE with MgSO_4_, C_18_, PSA, and GCB. The extract was dried and redissolved before analysis.	LC-MS/MS	[32]
		In the case of egg yolk, 2.5 g of homogenized sample + 2.5 mL of deionized water were mixed prior to extraction, performed as above. Powdered egg white was diluted to 12.3% w/v with deionized water, and a 5 g sample was extracted as above.		

Eggs, chicken, and pork	Phorate and its metabolites	Homogenized sample (5 g) was extracted with 15 mL of 1% AcOH in MeCN in the presence of MgSO_4_ and NaOAc; the supernatant was reextracted with 1% AcOH in MeCN, cleaned up with MgSO_4_, PSA, and C_18_. The extract was dried and redissolved before analysis.	UHPLC-MS/MS	[33]

Fish (tilapia and salmon)	Organochlorine pesticides and polychlorinated biphenyls	Homogenized fish (5 g) was extracted with 10 mL of Milli-Q water + 10 mL of MeCN in the presence of MgSO_4_, NaCl, Na_2_HCit·1.5H_2_O, and Na_3_Cit·2H_2_O. The supernatant was frozen and partitioned in CaCl_2_, followed by a second cleanup with MgSO_4_ and PSA.	GC-MS	[34]

Fish	Organochlorine pesticides	Homogenized fish (5 g) was extracted with MeCN (10 mL) in the presence of MgSO_4_ and NaCl, followed by d-SPE using PSA. Then, water and 1-undecanol were added, and the sample was cooled in an ice bath to separate the organic phase.	GC-MS	[35]

High-fat smoked salmon	Parent and substituted polycyclic aromatic hydrocarbons	Four QuEChERS extraction methods were tested. Optimized QuEChERS involved extraction with 2 : 2 : 1 (v/v/v) acetone/ethyl acetate/isooctane in the presence of AOAC or EN salts, followed by d-SPE with Sampli-Q AOAC fatty sample d-SPE tubes.	GC-MS	[36]

Fish muscle and liver tissues	Personal care products (biocides, synthetic musks, and benzotriazoles)	Homogenized fish muscle (2 g) or fish liver (0.5 g) was extracted with 1% AcOH in MeCN (10 mL) in the presence of MgSO_4_ and NaOAc, followed by d-SPE using PSA, MgSO_4_, and C_18_. The sample was evaporated and redissolved before analysis.	UPLC-MS/MS and GC-MS	[37]

Fish (carp and sturgeon)	Organochlorine and organophosphate pesticides	Homogenized fish muscle (5 g) was extracted with MeCN (10 mL) in the presence of Na_2_HCit·1.5H_2_O, Na_3_Cit·2H_2_O, NaCl, and MgSO_4_. A dual d-SPE cleanup was performed, using PSA + SAX + NH_2_, followed by C_18_ and CHCl_3_ addition and freezing-out to remove fats.	GC/Q-MS	[38]

Fish (catfish)	PCBs	Homogenized sample (3 g) was extracted with water (5 mL) and MeCN (30 mL) in the presence of MgSO_4_ and NaCl; d-SPE was performed with MgSO_4_, PSA, and C_18_.	GC-MS	[39]

Fish	Quinolones	Homogenized fish (5 g) was extracted with 5% HCOOH in MeCN (10 mL) in the presence of MgSO_4_, NaCl, NaOAc, and Na_2_HCit·1.5H_2_O, followed by d-SPE using C_18_ and MgSO_4_. The extract was dried and redissolved before analysis.	UHPLC-FLD	[40]

Fish	Organochlorine pesticides	Homogenized fish (5 g) was extracted with MeCN (5 mL) in the presence of MgSO_4_ and NaCl, followed by d-SPE with PSA + DLLME-SFO with 1-undecanol + water, and the sample was cooled in an ice bath to separate the organic phase.	GC-ECD	[41]

Bivalves	Pharmaceuticals	Frozen-dried sample (1 g) was mixed with 10 mL water and extracted with MeCN (10 mL) in the presence of EN salts, followed by cleanup with silica gel. The extract was dried and redissolved before analysis.	LC-MS/MS	[42]

Bivalves	Paralytic shellfish poisoning toxins	Homogenized sample (1 g) was extracted twice with 1% HCOOH (1 mL); protein was precipitated and d-SPE was performed using ABS Elut-NEXUS phase.	LC-MS	[43]

Honey	Nitrofuran metabolites and nitroimidazole	Honey (1 g) was extracted using 10 mL MeCN with MgSO_4_ and NaCl, without d-SPE. The extract was evaporated and redissolved before analysis.	LC-MS/MS	[44]

Honey	Pesticides	Honey (2.5 g) was heated in a water bath and extracted with 5 mL Na_2_EDTA + 5.0 mL MeCN in the presence of MgSO_4_ and NaCl, followed by d-SPE using PSA and MgSO_4._	GC-ECD	[45]

Honey	Pesticides	Honey (5 g) dissolved in 10 mL water was extracted with 10 mL 1% AcOH in MeCN:EtOAc (70 : 30), in the presence of MgSO_4_ and NaOAc, followed by d-SPE using MgSO_4,_ Florisil, and PSA.	UHPLC-MS/MS	[46]

Honey	Pesticides, PAHs, and PCBs	Honey (5 g) dissolved in 10 mL water was extracted with 10 mL MeCN in the presence of citrate salts, followed by d-SPE using PSA. Extracts were evaporated and redissolved; one part was directly analyzed by LC, whereas the other part was diluted with salted water and extracted with SPME for GC analysis.	LC-MS/MS, GC-MS/MS	[47]

Honey	Pyrrolizidine and tropane alkaloids	Homogenized honey (1.5 g) was dissolved with 0.1 M H_2_SO_4_ (10 mL), and Zn dust was added. After centrifugation, the sample was extracted with 10 mL MeCN in the presence of EN salts, followed by d-SPE with Q-Sep sorbent. The extract was dried and redissolved before analysis.	LC-HRMS	[48]
